# Use of Blood Donor Screening Data to Estimate Zika Virus Incidence, Puerto Rico, April–August 2016

**DOI:** 10.3201/eid2305.161873

**Published:** 2017-05

**Authors:** Michelle S. Chevalier, Brad J. Biggerstaff, Sridhar V. Basavaraju, M. Cheryl Bañez Ocfemia, Jose O. Alsina, Consuelo Climent-Peris, Robin R. Moseley, Koo-Whang Chung, Brenda Rivera-García, Melissa Bello-Pagán, Lisa L. Pate, Susan A. Galel, Phillip Williamson, Matthew J. Kuehnert

**Affiliations:** Centers for Disease Control and Prevention, Atlanta, Georgia, USA (M.S. Chevalier, S.V. Basavaraju, M.C. Bañez Ocfemia, R.R. Moseley, K.-W. Chung, M.J. Kuehnert);; Centers for Disease Control and Prevention, Fort Collins, Colorado, USA (B.J. Biggerstaff);; Banco de Sangre de Servicios Mutuos, San Juan, Puerto Rico (J.O. Alsina);; Banco de Sangre del Centro Medico of the Administración de Servicios Médicos, San Juan (C. Climent-Peris);; Puerto Rico Department of Health, San Juan (B. Rivera-García, M. Bello-Pagán);; Roche Molecular Systems, Inc., Pleasanton, California, USA (L.L. Pate, S.A. Galel);; Creative Testing Solutions, Tempe, Arizona, USA (P. Williamson)

**Keywords:** Zika virus, blood donors, Puerto Rico, incidence, arboviruses, viruses, vector-borne infections, flavivirus, mosquitoes

## Abstract

Puerto Rico has been heavily impacted by Zika virus, a mosquitoborne flavivirus that emerged in the Americas during 2015. Although most persons with Zika virus show no symptoms, the virus can cause neurologic and other complications, including fetal microcephaly. Local Zika virus transmission in Puerto Rico has been reported since December 2015. To prevent transfusion-associated transmission, local blood collection ceased in March 2016 but resumed in April 2016 after Zika virus screening of blood donations became available. Using data from screening of blood donations collected by the 2 largest blood centers in Puerto Rico during April 3–August 12, 2016, and assuming a 9.9-day duration of viremia, we estimated that 469,321 persons in Puerto Rico were infected during this period, for an estimated cumulative incidence of 12.9%. Results from blood donation screening during arboviral outbreaks can supplement routine clinical and surveillance data for improved targeting of prevention efforts.

Zika virus, a flavivirus transmitted primarily by *Aedes aegypti* mosquitoes, has rapidly spread in the Americas since it emerged in the region in 2015 (*1*). Although most infections are asymptomatic, Zika virus has been identified as a cause of adverse outcomes of pregnancy, including microcephaly and other congenital brain defects (*2*), and has been linked to Guillain-Barré syndrome (*3*) and severe thrombocytopenia (*4*,*5*). Zika virus also has been recognized as a potential threat to blood safety (*6*). In other arbovirus outbreaks, related mosquitoborne flaviviruses, such as West Nile virus and dengue virus, have been transmitted through blood transfusion; the high percentage of asymptomatic infections was a contributing factor (*7*). Retrospective nucleic acid testing (NAT) of blood donations after a large Zika virus outbreak in French Polynesia during 2013–2014 found detectable Zika virus RNA in 2.8% of blood donations (*8*), and cases of likely transfusion-transmitted Zika virus infection (through whole blood–derived platelets) were documented in Brazil (*9*,*10*).

Puerto Rico first reported local transmission of Zika virus in December 2015 (*11*) and has since been heavily affected. As of October 17, 2016, a total of 25,355 cases of locally acquired Zika virus infections had been reported from Puerto Rico to the Centers for Disease Control and Prevention (CDC) national arboviral surveillance system (ArboNET) (*12*).

To reduce the risk for transfusion-transmitted Zika virus infection, in February 2016, the Food and Drug Administration (FDA) recommended that all US areas with active Zika virus transmission cease blood collections unless donations are screened by NAT or treated with approved pathogen-reduction technology (*13*). Blood safety interventions in Puerto Rico were limited to importation of blood units from unaffected US areas and treatment of plasma and apheresis platelets with pathogen-reduction technology until early April 2016, when FDA authorized use of an individual donation NAT test (ID-NAT; cobas Zika, Roche Molecular Systems, Inc., Pleasanton, CA, USA) under an investigational new drug application (*14*).

Data from blood donor screening have been used during previous arbovirus outbreaks to supplement surveillance and guide the implementation of public health interventions. For example, in 2003, blood donor screening data were used to estimate the seasonal incidence of West Nile virus among the general US population (*15*). We describe the use of cobas Zika testing of blood donations from the 2 largest blood collection organizations in Puerto Rico to estimate the total number of incident Zika virus infections in Puerto Rico during April 3–August 12, 2016.

## Methods

Since April 3, 2016, all blood donations collected in Puerto Rico have been screened for Zika virus by using the cobas Zika ID-NAT, which uses PCR amplification to detect Zika virus RNA in plasma specimens. A blood donor with a reactive cobas Zika test result on initial donation is considered to be a presumptive viremic donor (PVD). In this study, we used data on PVDs to estimate Zika virus incidence.

For these analyses, we used data from blood donations collected by the Banco de Sangre de Servicios Mutuos (BSIS; San Jose, PR) during April 3–August 12, 2016, and by the Banco de Sangre del Centro Médico de la Administración de Servicios Médicos (ASEM; San Jose, PR) during April 4–July 31, 2016. These organizations collect most blood donations in Puerto Rico (*16*), with collections throughout the main island. Information collected and reported to CDC included a unique donor identification number, donor sex and age, city and ZIP code of donor residence, date of donation, and cobas Zika test result. City and ZIP code of donor residence were used to identify a donor’s municipality (i.e., county) and then health region as defined by the Puerto Rico Department of Health: Aguadilla, Arecibo, Bayamón, Caguas, Fajardo, Mayagüez, Metro/San Juan, and Ponce (*17*).

Because the minimum amount of time donors are required to wait between whole blood and plasma donations at blood centers is 56 and 28 days, respectively, the maximum number of donations per donor during the study period was 5. To estimate Zika virus incidence, all donations from any 1 donor were included in these analyses, except for repeat donations from donors who had a previous cobas Zika-reactive donation because such results could indicate infection and thus immunity. We also excluded donations from donors residing outside Puerto Rico.

To calculate the total number of incident Zika virus infections and the population incidence during the study period, we first calculated the proportions of cobas Zika-reactive donations to estimate the point incidence of Zika virus infection at the time of donation. The point incidence of cobas Zika-reactive donations, which we report aggregated to the week of collection, was then scaled to give estimates of Zika virus incidence during the referenced time frame. Estimates and 1-at-a-time 95% CIs of the number of incident Zika virus infections were computed weekly and cumulatively by week beginning April 3. The weekly values are estimates of the number of incident Zika virus infections during the given week; the weekly cumulative incidence values are aggregated estimates of the number of incident Zika virus infections from April 3 to the given week. The Zika virus incidence estimation process for April 3–August 12, 2016, followed the method of Busch et al. (*15*), although this approach was modified to incorporate the fact that donors are necessarily asymptomatic at time of donation. In brief, proportions of cobas Zika-reactive donations were multiplied by a factor given as the ratio of the duration of the period of collection to the average viremia duration, whereas Zika virus–infected persons are asymptomatic. Parameters used to characterize the average asymptomatic viremia duration were the overall average viremia duration, the average incubation period (i.e., duration from infection to symptom onset), and the proportion of asymptomatic infections. We used statistical computer simulation to account for uncertainty in these parameters.

Because demographic or geographic factors might have affected transmission rates across Puerto Rico, we compared the proportions of cobas Zika-reactive donations across these factors using Fisher exact test. Factors statistically significant at the 5% level were incorporated into the estimation procedure by simultaneously stratifying the donation and population data by these factors, using the procedure outlined earlier (Technical Appendix) to compute separate estimates of the numbers of incident Zika virus infections during the period of interest for each stratum and summing these values for an estimate of the total number of incident Zika virus infections. We divided this summation by the total size of the population at risk to give the estimated incidence of Zika virus infection for this population during the 5-month study period (Technical Appendix).

We used US Census estimates for 2014 for population totals by stratum (*18*). For the primary analyses, the estimates of the parameters used were 9.9 days (95% CI 6.8–21.6 days) for mean Zika virus viremia duration (*19*), 6.2 days (95% CI 5.3–7.1 days) for the mean Zika virus incubation period (*20*), and 0.79 (95% CI 0.73–0.90) for proportion asymptomatic (*8*). The key parameter was the mean duration of Zika virus viremia. We performed a sensitivity analysis to evaluate the influence of the specification of this parameter by computing estimates for the total number and percentage of Zika virus infections in the population for different values of Zika virus viremia duration, ranging from 7 to 21 days. Analyses were performed and graphics created in the R version 3.3.1 statistical software package (https://www.R-project.org/) by using purpose-written routines, and we used StatXact version Eleven (http://www.cytel.com) for Fisher exact test.

This study involved analyses of data collected as part of public health response activities. Therefore, the Office of the CDC Associate Director for Science considered it exempt from institutional review board review.

## Results

Data on 21,643 blood donors from BSIS and ASEM were reported to CDC for April 3–August 12, 2016. Of these donors, 21,468 (17,850 from BSIS and 3,618 from ASEM) were included in the analysis; 175 were excluded because of invalid data or residence outside of Puerto Rico. Included donors made 22,028 total blood donations during the study period. Of all included donors, 190 (153 BSIS and 37 ASEM) were PVDs; 21,278 had cobas Zika-nonreactive screening test results; 20,912 were first-time donors; and 14,407 (67%) were men (Table 1). Reported donor residence included all of the municipal health regions in Puerto Rico (Table 1). Among the 190 PVDs, 181 had reactive cobas Zika test results on their first donation, and 9 had nonreactive results at first donation but reactive results on repeat donation. Also among PVDs, 142 (75%) were men, 67 (35%) were 45–59 years of age, and 129 (68%) resided in either Metro/San Juan (44%) or Bayamón (24%) (Table 1). The overall rate of cobas Zika ID-NAT donor reactivity during the 5-month period was 89/10,000 donors.

**Table 1:** Characteristics of blood donors screened for Zika virus infection with cobas Zika ID-NAT at BSIS and ASEM, Puerto Rico, April 3–August 12, 2016*

Characteristic	Total donors screened, no. (%)	Presumptive viremic donors, no. (%)
Total	21,468 (100)	190 (100)
Sex		
M	14,407 (67.1)	142 (74.7)
F	7,061 (32.9)	48 (25.3)
Age at donation, y		
16–29	4,313 (20.1)	39 (20.5)
30–44	7,179 (33.4)	51 (26.8)
45–59	7,046 (32.8)	67 (35.3)
60–74	2,751 (12.8)	31 (16.3)
>75	179 (0.8)	2 (1.1)
Month of donation		
April	4,339 (20.2)	14 (7.4)
May	4,891 (22.8)	33 (17.4)
June	5,602 (26.1)	67 (35.3)
July	4,773 (22.2)	56 (29.5)
August	1,863 (8.7)	20 (10.5)
Region of residence		
Aguadilla	751 (3.5)	9 (4.7)
Arecibo	1,883 (8.8)	15 (7.9)
Bayamón	4,797 (22.3)	45 (23.7)
Caguas	4,359 (20.3)	19 (10.0)
Fajardo	979 (4.6)	2 (1.1)
Mayagüez	917 (4.3)	2 (1.1)
Metro/San Juan	6,081 (28.3)	84 (44.2)
Ponce	1,701 (7.9)	14 (7.4)
Donation type		
First-time	20,912 (97.4)	168 (88.4)
Repeat	556 (2.6)	22 (11.6)

*Data for August 1–August 12, 2016 available only for BSIS. cobas Zika, Roche Molecular Systems, Inc., Pleasanton, CA, USA. ASEM, Banco de Sangre del Centro Médico de la Administración de Servicios Médicos; BSIS, Banco de Sangre de Servicios Mutuos; ID-NAT, individual nucleic acid testing.

Combining donation data from all health regions, we found no statistically significant difference in cobas Zika test reactivity by age group (p = 0.32), but the proportion of reactivity (number of reactive donations/number of donations) significantly differed by donor sex (women, 48 [0.67%] of 7,125; men, 142 [0.95%] of 14,903; risk ratio 1.41, 95% CI 1.02–1.96; p = 0.036) and by health region (p<0.001). By health region, the association between reactivity and sex was significant in only 1 (Ponce, in which all of the 14 reactive donations were from men).

**Figure 1:**
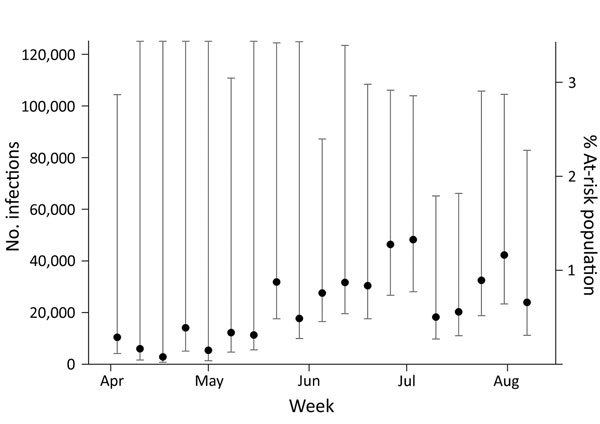
Individual weekly estimates of the number and percentage of at-risk population with incident Zika virus infections computed with cobas Zika (Roche Molecular Systems, Inc., Pleasanton, CA, USA) individual nucleic acid testing results from Banco de Sangre de Servicios Mutuos and Banco de Sangre del Centro Médico de la Administración de Servicios Médicos, Puerto Rico, April 3–August 12, 2016. These estimates assume a mean viremia duration of 9.9 days (SD ± 3.9). To retain readability of the point estimates, some of the confidence interval line segments extend beyond the vertical boundary. Data for August 1–August 12, 2016 available only for Banco de Sangre de Servicios Mutuos. Error bars indicate 95% CIs.

**Figure 2:**
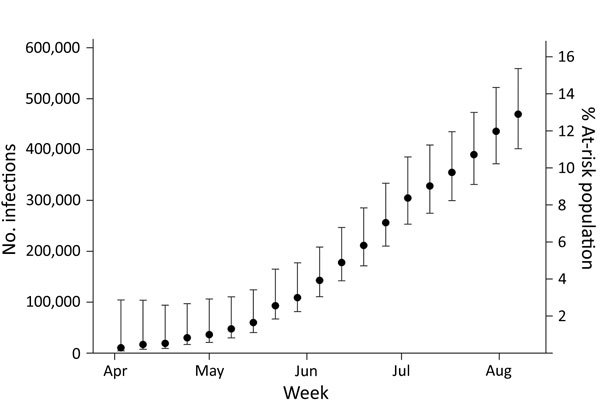
Cumulative weekly estimates of the number and percentage of at-risk population with incident Zika virus infections computed with cobas Zika (Roche Molecular Systems, Inc., Pleasanton, CA, USA) individual nucleic acid testing results from Banco de Sangre de Servicios Mutuos and Banco de Sangre del Centro Médico de la Administración de Servicios Médicos, Puerto Rico, April 3–August 12, 2016. These estimates assume a mean viremia duration of 9.9 days (SD ± 3.9). Each weekly estimate is computed from all donation data collected from April 3 to the given week. Data for August 1–August 12, 2016 available only for Banco de Sangre de Servicios Mutuos. Error bars indicate 95% CIs.

Based on the 2014 US Census Puerto Rico population estimate of 3,639,000 residents and using a mean viremia duration of 9.9 days (SD ± 3.94 days) and stratifying by health region and sex, we estimated the number of incident Zika virus infections for April 3–August 12, 2016, to be 469,321 (95% CI 401,477–559,126). This number represents a Zika virus cumulative incidence of 12.9% (95% CI 11.0%–15.4%) for Puerto Rico for the 5-month period (Figures 1, 2). The estimated number of Zika virus infections for reproduction-aged women (16–44 years) was 69,675 (95% CI 48,226–117,578), which represents 9.7% (95% CI 6.7%–16.3%) of the total population of women of reproduction age in Puerto Rico.

**Figure 3:**
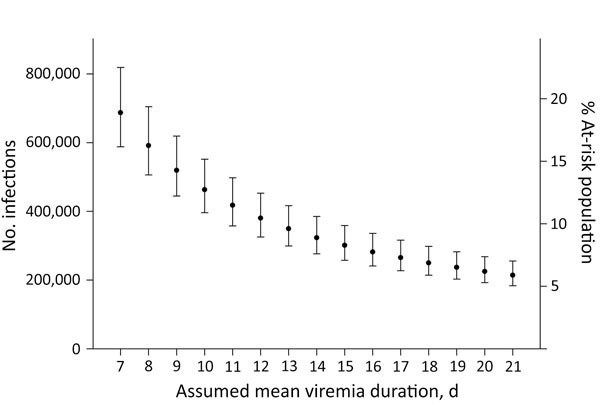
Estimated total number of incident Zika virus infections and percentage of the at-risk population infected with Zika virus during the study period by assumed mean viremia duration computed with cobas Zika (Roche Molecular Systems, Inc., Pleasanton, CA, USA) individual nucleic acid testing results from Banco de Sangre de Servicios Mutuos and Banco de Sangre del Centro Médico de la Administración de Servicios Médicos, Puerto Rico, April 3–August 12, 2016. Data for August 1–August 12, 2016 available only for Banco de Sangre de Servicios Mutuos. Error bars indicate 95% CIs.

Estimates of the total number and percentage of the population infected with Zika virus during the study period are given using mean viremia durations of 7–21 days (Table 2, Figure 3) Estimates for percentage of the population infected with Zika virus declined with increasing viremia duration, ranging from 16.1% for 7 days viremia duration to 5.9% for 21 days. The incidence estimate would be lower if we had used an estimated mean viremia duration of >9.9 days in our calculations (Figure 3).

**Table 2:** Sensitivity analysis for Zika virus infections, computed from BSIS and ASEM cobas Zika ID-NAT results, Puerto Rico, April 3–August 12, 2016*

Mean viremia duration, d	Total no. infections (95% CI)	Population infected, % (95% CI)
7	684,937 (585,924–816,000)	18.82 (16.1–22.43)
14	323,525 (276,756–385,431)	8.89 (7.61–10.59)
21	214,563 (183,546–255,619)	5.89 (5.04–7.02)

*Data for August 1–August 12, 2016 available only for BSIS. cobas Zika, Roche Molecular Systems, Inc., Pleasanton, CA, USA. ASEM, Banco de Sangre del Centro Médico de la Administración de Servicios Médicos; BSIS, Banco de Sangre de Servicios Mutuos; ID-NAT, individual nucleic acid testing.

## Discussion

In this analysis of routine blood donation screening data from the 2 largest blood collection centers in Puerto Rico, we estimated that 469,321 persons were infected with Zika virus during April–August 2016, assuming a mean viremia duration of 9.9 days. The estimated cumulative incidence of Zika virus infection for the study period was 12.9%.

Among the parameters used in this estimation, mean duration of Zika virus viremia is most influential because it is inversely related to the overall estimate of the number of persons infected with Zika virus in Puerto Rico. To our knowledge, the mean duration of viremia in serum is still unknown but has been shown to range from 4–10 weeks in gravid women (*21*) to 3–18 days in asymptomatic, nonpregnant persons (*19*). We used the value of 9.9 days (95% CI 6.8–21.6 days) on the basis of a literature review of 25 cases that provided doubly interval-censored data (*19*). The wide 95% CI for the mean viremia duration estimates reflected the current paucity of data on viremia duration. To evaluate the influence of this key parameter in our analyses, we included a sensitivity analysis by varying the assumed mean viremia duration and computing corresponding incidence estimates of Zika virus infection.

Using the mean viremia duration of 9.9 days gave a substantially higher total number of incident Zika virus infections than the number of new laboratory-confirmed infections reported from Puerto Rico to ArboNET during the same period (≈10,000 infections) (*22*). However, because of limitations in general population testing, this system reflects only symptomatic persons and a subset of asymptomatic pregnant women. One advantage of using blood donor screening as a surveillance tool is that it can rapidly capture real-time, cumulative incidence data from a large, diverse convenience sample of the general population; this information might otherwise be unattainable during a public health emergency. As observed during previous outbreaks of arbovirus diseases (e.g., West Nile, dengue, chikungunya) in the continental United States and territories, blood donation screening conducted during outbreaks can identify persons who are acutely infected and asymptomatic, which can aid in active case surveillance and enable characterization of viral and immunologic dynamics of clinical illness (*15*,*23*,*24*). Detection of Zika virus–infected asymptomatic blood donors is important not only for preventing transfusion-transmitted infections but also because the infection can be sexually transmitted and might result in adverse birth outcomes, even among pregnant women who do not have signs or symptoms. As US blood centers implement updated FDA recommendations for universal Zika virus blood donation screening (*25*), the coupling of prompt communication of reactive blood donor screening results to public health authorities with appropriate prevention messages and other public health interventions will become increasingly important in helping to mitigate the spread of Zika virus.

The findings of this study are subject to several limitations. First, the number of persons residing in Puerto Rico (estimated at 3.4 million in 2016 by the Puerto Rico Department of Health) might differ from the 2014 US Census population estimate of 3.6 million in our model. Second, the demographic composition of blood donors, specifically sex and age, does not match that of the general population. Data from this study show that men represented >67% of blood donors. Furthermore, data from persons <16 years of age were unavailable because of blood donor age restrictions, so the estimates we give for the whole population include an extrapolation to this age group. Although the data do not indicate a substantial difference in Zika virus incidence by age, whether the lack of data from the 0–15-year age group substantially affected our population incidence estimates is unknown. Alternatively, with regard to sex and infectivity, few data are available to support a predisposition for Zika virus infection in men; nevertheless, the statistically significant study finding of a male-to-female ratio of infectivity of 1.41 among donors suggests the need for further exploration of any possible interplay between sex and the length of viremia from Zika virus infection or Zika virus susceptibility. Third, blood donors are subjected to a medical examination and questionnaire to ascertain signs and symptoms of illness, and potential donors who are feeling ill are excluded from donation. Consequently, blood donor screening data might underestimate infection incidence because of the exclusion of symptomatic persons. Because our model adjusted for the exclusion of these persons, this limitation should not affect our analysis; however, this factor is an important consideration when blood screening data are used as a surveillance tool. Last, the duration of Zika virus viremia is unknown, and assumptions made for this model were based on limited data. Important research priorities will be to determine viremia duration through longitudinal follow-up of infected blood donors and studies of acute infection in animal models, resulting in more precise calculation of viral kinetics.

In summary, the findings of this study suggest that a much larger proportion of the population in Puerto Rico was infected with Zika virus during April–August 2016 than reported through surveillance. Although Puerto Rico mandates reporting of Zika virus infections, the conveyance of arboviral surveillance data across local, state, and national levels is often delayed and can affect strategic planning and interventions. Blood donation screening data can augment clinical Zika virus surveillance data to provide real-time communication of Zika virus incidence estimates to enable better ascertainment of the extent of outbreaks and improved targeting of prevention and response efforts.

Technical Appendix Additional statistical methods for study of Zika virus infection incidence in Puerto Rico using blood donor data.
