# Supplementary material for: Use of Blood Donor Screening Data to Estimate Zika Virus Incidence, Puerto Rico, April–August 2016
Source: Emerg Infect Dis. 2017 May;23(5):790–5. doi: 10.3201/eid2305.161873 (PMC5403024; doi:10.3201/eid2305.161873)
Supplement: Technical Appendix — Additional statistical methods for study of Zika virus infection incidence in Puerto Rico using blood donor data. [file 16-1873-Techapp-s1.pdf]

Article DOI: http://dx.doi.org/10.3201/eid2305.161873

Use of Blood Donor Screening Data to Estimate Zika Virus Incidence, Puerto Rico, 2016

Technical Appendix

Additional Statistical Methods

For estimation of both the numbers of incident infections and incidence during a time frame of interest, blood donations were stratified by health region and sex of donor, resulting in 16 strata. We assumed that donors were as likely as the general population to be infected with Zika virus and then to develop illness.

Let N_{sk} be the number of blood donations tested for Zika virus in stratum $s = 1,2,\dots,S = 16$ during week $k \in \{1,2,\dots,K = 19\}$, and let Z_{sk} be the corresponding number of those that were "viremic" (i.e., cobas Zika reactive). For each $s = 1,2,\dots,S$, let

$$P_{s;w_1,w_2} = \frac{\sum_{k=w_1}^{w_2} Z_{sk}}{\sum_{k=w_1}^{w_2} N_{sk}} \equiv \frac{Z^+_{s;w_1,w_2}}{N^+_{s;w_1,w_2}}$$

be the proportion of cobas Zika reactive donations in stratum s for weeks w_1 through w_2, inclusive. Analyses presented include weekly ($w_1 = w_2$ for each week $k \in \{1,2,\dots,K = 19\}$) and cumulative weekly ($w_1 = 1, w_2 \in \{1,2,\dots,K = 19\}$) estimates.

In the absence of historical information on the numbers of weekly donations by donors' health region and sex, so that we might model the N_{sk}, we assume throughout that these numbers are fixed.

To ease notation, take the weeks w_1 and w_2 as fixed for a particular time frame of interest, and use the simplified notation N_s^+, Z_s^+, $P_s = Z_s^+/N_s^+$ for corresponding stratum sums, and sample proportions for the time frame of interest. Let T_s be the stratum-specific population sizes, obtained from available US Census data (http://www.census.gov), so that $T = \sum_{s=1}^{S} T_s$ is

the total population size. Before accounting for the transient nature of viremia, a crude estimate of the number of infections during the time period of interest is then

$$I_{crude} = \sum_{s=1}^{S} T_s \, P_s$$

leading to a crude estimate of the proportion of the population infected over the time period of interest of

$$\Pi_{crude} = \frac{I_{crude}}{T - I_{crude}^{<}} = \frac{\sum_{s=1}^{S} T_s \, P_s}{\sum_{s=1}^{S} T_s - \left(\sum_{s=1}^{S} T_s P_s \right)^{<}}$$

where the superscript $<$ indicates these values are computed on the whole time period before the one used in the given estimation. That is, if I_{crude} is an estimate of the number of infections for the time period w_1 through w_2, then $I_{crude}^{<}$ is an estimate of the number of infections for the time period 1 through $w_2 - 1$. The denominator of Π_{crude} is an estimate of the size of the at-risk population, taking the total population size and subtracting from the estimated number of infections up to the time period of interest.

We next compute an associated crude $100(1-\alpha)\%$ CI for the number of Zika virus infections in the whole population (over w_1 through w_2) adapting the methods of Yan and Su (*1*) as follows. Letting Φ be the standard normal CDF, compute

$$\zeta = \Phi(1-\alpha/2) \frac{\sqrt{\sum_{s=1}^{S} T_s^2 \frac{P_s(1-P_s)}{N_s^+}}}{\sum_{s=1}^{S} T_s \sqrt{\frac{P_s(1-P_s)}{N_s^+}}}$$

A crude lower (-) and upper (+) confidence interval, (L_{crude}, U_{crude}), for the total number of Zika virus infections (over weeks w_1 to w_2) is then

$$(L_{crude}, U_{crude}) = \sum_{s=1}^{S} T_s \left(\frac{P_s + \zeta^2/(2N_s^+)}{1 + \zeta^2/N_s^+} \mp \frac{\zeta}{1 + \zeta^2/N_s^+} \sqrt{\frac{P_s(1-P_s)}{N_s^+} + \frac{\zeta^2}{4N_s^{+2}}} \right)$$

which are the respective weighted sums of the stratum-specific Wilson (1927) score confidence intervals using the normal quartile ζ, which has been adjusted for relative stratum-specific variation. The crude CI for the proportion of infections is obtained as with Π_{crude}, by dividing the limits L_{crude} and U_{crude} by $\sum_{s=1}^{S} T_s - I_{crude}^{<}$.

To account for the transient nature of viremia (here, cobas Zika positivity, or RNA-positivity) in determining infection, we follow Busch et al. (2006) and scale the estimate (and CI) of the number of infections by a factor representing the relative likelihood of a donor being viremic (here, cobas Zika reactive) at donation. Specifically, let d be the duration of collection (e.g., $d = 21$ days), and let $\overline{v}$ be the mean duration of Zika virus viremia, a value for which is specified from an exogenous source. The factor used by Busch et al., was $f_B = d/\overline{v}$. Persons who are ill, with symptomatic infection, are deferred from donation either by self-deferral or by point-of-donation screening; f_B does not account for this, introducing a bias. (This observation was mentioned in the discussion in Busch et al.) We therefore modify this factor to reflect that only asymptomatic persons are donors. Let p_a be the proportion of infections that are asymptomatic, and let $\overline{i}$ be the mean incubation period, that is, the duration from infection (actually, the commencement of viremia here) to symptom onset (for symptomatic infections). Then the mean duration of viremia for asymptomatic persons (including those who go on to develop symptoms after a period of infection and viremia) is $\overline{v}p_a + \overline{i}(1 - p_a)$. The ratio $\overline{v}/[\overline{v}p_a + \overline{i}(1 - p_a)]$ therefore represents a factor to correct f_B for its bias, giving

$$f = f_B \frac{\overline{v}}{\overline{v}p_a + \overline{i}(1 - p_a)} = \frac{d}{\overline{v}p_a + \overline{i}(1 - p_a)}$$

as a factor to account for the likelihood that an infected donor donates while viremic, but using the correct mean duration of viremia for donors.

Accounting for the transient viremia, we then adjust the total number of Zika virus infections in the population during the time period of interest as $I_{adj} = f\, I_{crude}$, and the associated CI is also scaled, $(L_{adj}, U_{adj}) = (f\, L_{crude}, f\, U_{crude})$.

As noted, we use exogenous information to obtain numerical values for $\overline{v}$, $\overline{i}$, and p_a. These values are obtained from published literature and clearly are uncertain. To account for this uncertainty in our estimation, we compute final estimates by simulating the distribution for f

under modeling assumptions for the parameter estimates, and using the mean of this distribution as the adjustment factor. For $j = 1,2, \ldots, J = 10{,}000$ simulations, we sample values for these parameters from the following distributions:

$$\begin{aligned} \bar{v}^{(j)} &\sim \text{Weibull with mean } 9.97 \text{ and variance } 15.54 \\ \bar{i}^{(j)} &\sim \text{normal with mean } 6.20 \text{ and variance } 0.23 \\ p_a^{(j)} &\sim \text{beta with mean } 0.79 \text{ and variance } 0.0019 \end{aligned}$$

With these values, write $f^{(j)}$ as simulated values of f. The mean of the distribution of f (viewed as a random variable when estimates are used for the parameters) is then estimated as $\bar{f} = \frac{1}{J}\sum_{j=1}^{J} f^{(j)}$. We then computed the final estimate I as

$$I = \bar{f}\, I_{adj} = \frac{1}{J}\sum_{j=1}^{J} f^{(j)}\, I_{adj} = \frac{d}{J}\sum_{j=1}^{J} \frac{I_{adj}}{\bar{v}^{(j)} p_a^{(j)} + \bar{i}^{(j)}(1 - p_a^{(j)})}$$

with confidence intervals computed analogously as $(L, U) = \left(\bar{f} L_{adj}, \bar{f} U_{adj}\right)$. The final estimate of the proportion infected over the time of interest, $\Pi = I/(T - I^{<})$, and associated CI $\left(L/(T - I^{<}), U/(T - I^{<})\right)$ are then computed.

Reference

1. Yan X, Su XG. Stratified Wilson and Newcombe confidence intervals for multiple binomial proportions. Stat Biopharm Res. 2010;2:329–35. http://dx.doi.org/10.1198/sbr.2009.0049
